# CDC Grand Rounds: Preventing Youth Violence

**Published:** 2015-02-27

**Authors:** Corinne David-Ferdon, Thomas R. Simon, Howard Spivak, Deborah Gorman-Smith, Sheila B. Savannah, Robert L. Listenbee, John Iskander

**Affiliations:** 1 Division of Violence Prevention, National Center for Injury Prevention and Control, CDC; 2 National Institute of Justice, US Department of Justice; 3 University of Chicago; 4 Houston Department of Health and Human Services; 5 Office of Juvenile Justice and Delinquency Prevention, US Department of Justice; 6 Office of the Director, CDC

Youth violence occurs when persons aged 10–24 years, as victims, offenders, or witnesses, are involved in the intentional use of physical force or power to threaten or harm others. Youth violence typically involves young persons hurting other young persons and can take different forms. Examples include fights, bullying, threats with weapons, and gang-related violence. Different forms of youth violence can also vary in the harm that results and can include physical harm, such as injuries or death, as well as psychological harm. Youth violence is a significant public health problem with serious and lasting effects on the physical, mental, and social health of youth. In 2013, 4,481 youths aged 10–24 years (6.9 per 100,000) were homicide victims (1). Homicide is the third leading cause of death among persons aged 10–24 years (after unintentional injuries and suicide) and is responsible for more deaths in this age group than the next seven leading causes of death combined (Figure) (1). Males and racial/ethnic minorities experience the greatest burden of youth violence. Rates of homicide deaths are approximately six times higher among males aged 10–24 years (11.7 per 100,000) than among females (2.0). Rates among non-Hispanic black youths (27.6 per 100,000) and Hispanic youths (6.3) are 13 and three times higher, respectively, than among non-Hispanic white youths (2.1) (1). The number of young persons who are physically harmed by violence is more than 100 times higher than the number killed. In 2013, an estimated 547,260 youths aged 10–24 years (847 per 100,000) were treated in U.S. emergency departments for nonfatal physical assault–related injuries (1).

Data from death certificates and emergency departments help communities understand and prevent youth violence but reflect only part of the problem. According to CDC’s 2013 Youth Risk Behavior Survey, one in four high school students reported being in at least one physical fight in the past year, and 17.9% reported that they carried a weapon (gun, knife, or club) at least once in the past 30 days (2). Youth violence also occurs in schools and harms students’ ability to participate fully in school life. In 2013, 19.6% of high school students reported being bullied at school in the past year, 6.9% said they were threatened or injured with a weapon on school property in the past year, and 7.1% reported missing at least 1 day of school in the past 30 days because they felt unsafe either at school or on their way to or from school (2).

Youths who are victims of violence are at greater risk for many other physical and mental health problems and other difficulties, including smoking, obesity, high-risk sexual behavior, depression, academic difficulties, and suicide (3–5). The impact of youth violence extends beyond the young perpetrators and victims to affect entire communities. Each year, youth homicides and nonfatal physical assault-related injuries result in an estimated $19.5 billion in combined medical and lost productivity costs (1). Violence can increase health care costs, decrease property values, and disrupt social services (6).

## Evidence-Based Youth Violence Prevention

Youth violence is not inevitable. It is preventable. Research supported by CDC and other groups has identified many approaches that contribute to significant reductions in youth violence and other risk behaviors and significant cost savings (7,8). Most effective prevention approaches work by modifying individual-level (e.g., problem-solving, communication, anger management skills) or relationship-level (e.g., parental supervision, consistent discipline, and communication skills) factors. A growing body of research shows the prevention potential of approaches that modify community-level factors (e.g., physical environments, norms about violence).

Universal school-based prevention programs are the most common approach to youth violence prevention. In general, these programs change how youths think and feel about violence and develop skills to avoid violence and resolve disputes nonviolently. These programs are designed to reach all students in a given school or grade. A systematic review of 53 studies by the Community Preventive Services Task Force (Community Guide) found a median reduction in violent behavior of 15% (9). These programs can be effective in different school environments, regardless of socioeconomic status, crime rate, or predominant ethnicity of students. One example is Life Skills Training, a 30-session curriculum that teaches students self-management and social skills (8). Life Skills Training has resulted in lower rates of violence, delinquency, and other high risk behavior. In the state of Washington, cost-benefit analyses suggest a savings of $11.58 for each dollar spent on Life Skills Training (10).

Parenting and family-focused strategies also have been extensively researched. These prevention approaches provide caregivers with support and teach communication, problem-solving, monitoring, and behavior management skills (8). Some are designed for youths with known histories of difficulties (e.g., chronic offenders, abuse victims) and include intensive services to address individual, family, school, and community factors that contribute to violence and delinquency. One example is therapeutic foster care for chronically delinquent juveniles, which involves placing at-risk youths with specially trained foster families for several months to provide a structured environment and intensive services. The Community Guide reviewed this approach and found it to reduce violent crime by 71.9% among participants when compared with youths in standard group residential treatment facilities and to have net benefits of $20,351 to $81,664 per youth (9).

Economic, policy, and violence prevention strategies that address community-level factors are emerging. One promising approach is business improvement districts, which are public-private partnerships that invest resources in local services and activities (e.g., street cleaning, security, adding parks and other green spaces) to increase the appeal and use of an area. A CDC-supported evaluation of 30 business improvement districts in Los Angeles found a 12% drop in robberies, an 8% drop in violent crime, and substantial economic benefits (e.g., savings from the decline in robbery alone offsetting implementation costs) (11). Growing research suggests other community-level approaches, such as street outreach and mobilization activities (e.g., Ceasefire and Safe Streets), can result in significant reductions in youth violence (8).

## Challenges to Preventing Youth Violence

Many communities are not aware of, or are unable to take advantage of, the strategies known to reduce youth violence. CDC’s Striving to Prevent Youth Violence Everywhere (STRYVE) initiative strengthens community capacity and collaboration to strategically select and implement evidence-based strategies (7,12). STRYVE resources are designed to help communities identify and access evidence-based youth violence prevention programs, policies, and practices. Examples of resources used by STRYVE and other CDC youth violence technical assistance activities include CDC’s *Preventing Youth Violence: Opportunities for Action* and its companion guide, CDC’s *STRYVE Online Strategy Selector Tool*, and the Office of Juvenile Justice and Delinquency Prevention’s *Model Programs Guide* (8,12,13).

No one program, practice, or policy can address all the factors that contribute to youth violence. Research conducted by CDC’s Youth Violence Prevention Centers (YVPCs) shows that a comprehensive prevention approach that includes multiple strategies to address individual, relationship, and community-level risk factors is critical. For example, work by the Chicago YVPC demonstrates that comprehensive public health approaches can have a broader reach and more sustained effects than the implementation of a single prevention program. Preliminary data suggest that between 2010 and 2013 there was a 50% decline in homicides in a Chicago community using comprehensive strategies (e.g., Schools and Families Educating Children, GREAT Families, and Ceasefire) (14). The decline in homicides and other outcomes (e.g., violent crime) was the result of the synergistic effect of all the implemented strategies and not the result of a single strategy (14).

Collaboration between governmental and nongovernmental groups can facilitate sustained preventive action. Government partners at federal, state, and local levels include justice, education, labor, social services, medical and mental health, and public health and safety. Community partners, such as businesses, youth-serving organizations (e.g., YMCA and Boys and Girls Clubs), and faith-based institutions as well as community residents are also critical. Additionally, young persons offer important perspectives. For example, young persons play an integral role in the prevention approaches implemented by CDC’s STRYVE-funded Houston Health Department and its partners. Surveys with youths and conducted by youths demonstrated that Houston youths felt disconnected from their community and neighbors, wanted to find ways to improve their community, and had few structured out-of-school activities (14). Houston partners used this information, other data sources, and CDC technical assistance to develop a comprehensive prevention plan. They selected evidence-based approaches, such as Youth Empowerment Solutions (e.g., structured opportunities for youth to work with adults and organizations to change social and physical environments) and principles of Crime Prevention through Environmental Design (e.g., maintenance and management of community space such as parks and increased lighting), to enhance the ability of neighborhood organizations to engage youth in violence prevention activities.

Another surmountable challenge is a tendency for communities to wait to intervene following violence rather than proactively preventing it from starting. Partners from the Department of Justice are increasingly focusing on the need for a collaborative, comprehensive approach that includes prevention. Robert L. Listenbee, Administrator of the Office of Juvenile Justice and Delinquency Prevention, stresses that communities cannot arrest their way out of the problem of youth violence and notes that a broader, more nuanced approach is required (14). Most youths in the juvenile justice system have been exposed to trauma (e.g., abuse and community violence), thus a part of reducing youth violence is providing these youths with services to help them recover. Judicial, medical, and social interventions that are implemented after youth violence occurs can help stop violence from continuing or progressing by addressing or ameliorating some of the consequences of violence (e.g., physical and mental trauma) that might increase the likelihood violence could occur again (8). Although important, these responses are not sufficient because they do not stop violence before it starts. Some interventions even increase the risk for subsequent violence. For example, the Community Guide systematic review of the transfer of juvenile offenders to adult criminal courts showed this strategy resulted in a 34% increase in rearrests for violent crimes (9). Primary prevention must be part of the collection of approaches communities use. Multi-agency collaborations, such as the National Forum on Youth Violence Prevention, are more commonly noting that youth violence is a public health problem and not just a law enforcement issue (15). Youth violence can be addressed by encouraging communities to use a data-driven, balanced approach that includes prevention, intervention, enforcement, and community reintegration following detention strategies (15).

## Public Health Role in Preventing Youth Violence

Preventing youth violence has far reaching benefits for health, safety, and economic development. The prevention of youth violence can lower morbidity and mortality from injuries and has the potential for reducing risks for other health problems, such as alcohol and substance abuse, obesity, and chronic diseases, and can result in cost savings for the justice, education, and health care systems. Public health professionals have a clear responsibility to help reduce the health burden of youth violence. With its emphasis on a science-driven approach, the public health sector brings a clear focus on prevention and the promotion of population-wide health, safety, and well-being. The public health community has the skills and expertise to collect and analyze relevant data, select and implement comprehensive prevention strategies, and organize and integrate efforts of diverse partners to successfully address the complex health issues of youth violence (8).

## Figures and Tables

**FIGURE:**
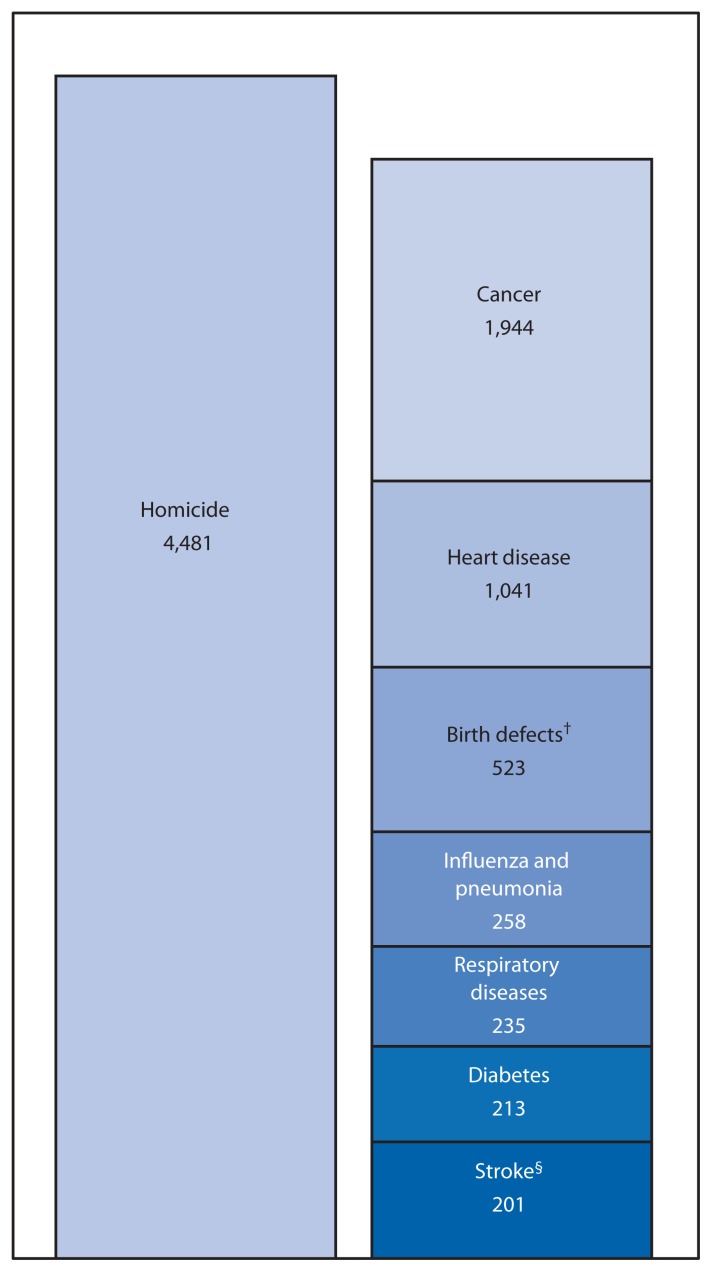
Third leading cause of death (homicide) among persons aged 10–24 years compared with the fourth through tenth leading causes of death in the same age group — United States, 2013* * Does not include the two leading causes of death among persons aged 10–24 years in 2013: unintentional injuries (12,394 deaths) and suicide (5,264). ^†^ Congenital anomalies. ^§^ Cerebrovascular diseases.

## References

[1] CDC Web-based Injury Statistics Query and Reporting System (WISQARS).

[2] CDC (2013). Youth online: high school YRBS.

[3] Arseneault L, Walsh E, Trzeniewski K, Newcombe R, Caspi A (2006). Bullying victimization uniquely contributes to adjustment problems in young children: a nationally representative cohort study. Pediatrics.

[4] Finkelhor D, Turner H, Ormrod R (2006). Kid’s stuff: the nature and impact of peer and sibling violence on younger and older children. Child Abuse Negl.

[5] Menard S (2002). Short- and long-term consequences of adolescent victimization.

[6] Mercy J, Butchart A, Farrington D, Cerdá M, Krug EG, Dahlberg LL, Mercy JA, Zwi AB, Lozano R (2002). Youth violence. World report on violence and health.

[7] CDC (2014). Injury prevention and control: youth violence: national centers for excellence in youth violence prevention.

[8] David-Ferdon C, Simon TR (2014). Preventing youth violence: opportunities for action.

[9] Community Preventive Services Task Force (2014). Community guide: violence prevention.

[10] Washington State Institute for Public Policy Cost-benefits results.

[11] MacDonald J, Golinelli D, Stokes RJ, Bluthenthal R (2010). The effect of business improvement districts on the incidence of violent crimes. Inj Prev.

[12] CDC (2014). Striving to reduce youth violence everywhere online.

[13] Office of Justice Programs (2014). Model programs guide.

[14] Spivak H, Gorman-Smith D, Savannah S, Listenbee RL (2014). CDC public health grand rounds: preventing youth violence.

[15] National Forum on Youth Violence Prevention Interagency Working Group on Youth Programs.

